# *erythro*-{1-Bromo-1-[(1-phenyl­eth­yl)sulfon­yl]eth­yl}benzene

**DOI:** 10.1107/S2414314624001895

**Published:** 2024-03-06

**Authors:** Peter W. R. Corfield

**Affiliations:** a Department of Chemistry, Fordham University, 441 East Fordham Road, Bronx, NY 10458, USA; Vienna University of Technology, Austria

**Keywords:** crystal structure, sulfone, diasteromer, 1,3-elimination, C—H⋯O and C—H⋯Br hydrogen bonding

## Abstract

The title compound, C_16_H_17_BrO_2_S, crystallizes as the *erythro* (*RR/SS*) isomer of a pair of sulfones that were diastereomeric due to chirality of the α-carbon atoms on the sulfone sulfur atom. The structural analysis was pivotal in showing that the 1,3 elimination reactions of these compounds, which lead to substituted stilbenes, occur with inversion at each asymmetric carbon atom. In the crystal, C—H⋯Br and C—H⋯O hydrogen bonds link the mol­ecules into a tri-periodic inter­molecular network.

## Abstract 2

Structural analysis of the title diasteromeric sulfone determines this to be the *erythro* (*RR/SS*) isomer, and was pivotal in showing that the 1,3-elimination reactions of these compounds, which lead to substituted stilbenes, occur with inversion at each asymmetric carbon atom.

## Graphical abstract

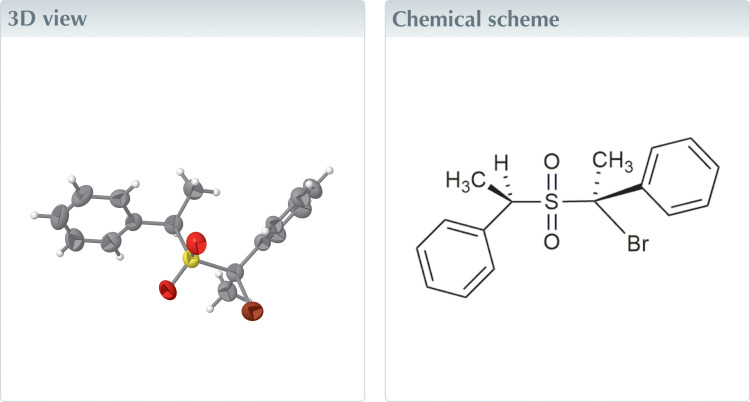

## Structure description

In an earlier paper (Bordwell *et al.*, 1970 ▸), we described how two mono­bromo sulfone diastereomers with melting points of 349 and 385 K had been prepared. The final products from a Ramberg–Bäcklung reaction on these compounds were primarily *cis-α,α*’-di­methyl­stilbene for the higher melting stereoisomer, and *trans-α,α*’-di­methyl­stilbene for the lower melting isomer. The crystal-structure determination of the title compound, which is the higher melting isomer, enabled the determination that the reactions involved inversion at each of the asymmetric α-C atoms, but no crystallographic details were given in the above paper. Continuing inter­est in the stereochemistry of such reactions (Düfert, 2023 ▸; Paquette, 2001 ▸) prompted this publication to give details of the structure analysis of the title compound, C_16_H_17_BrO_2_S.

**Figure 1:**
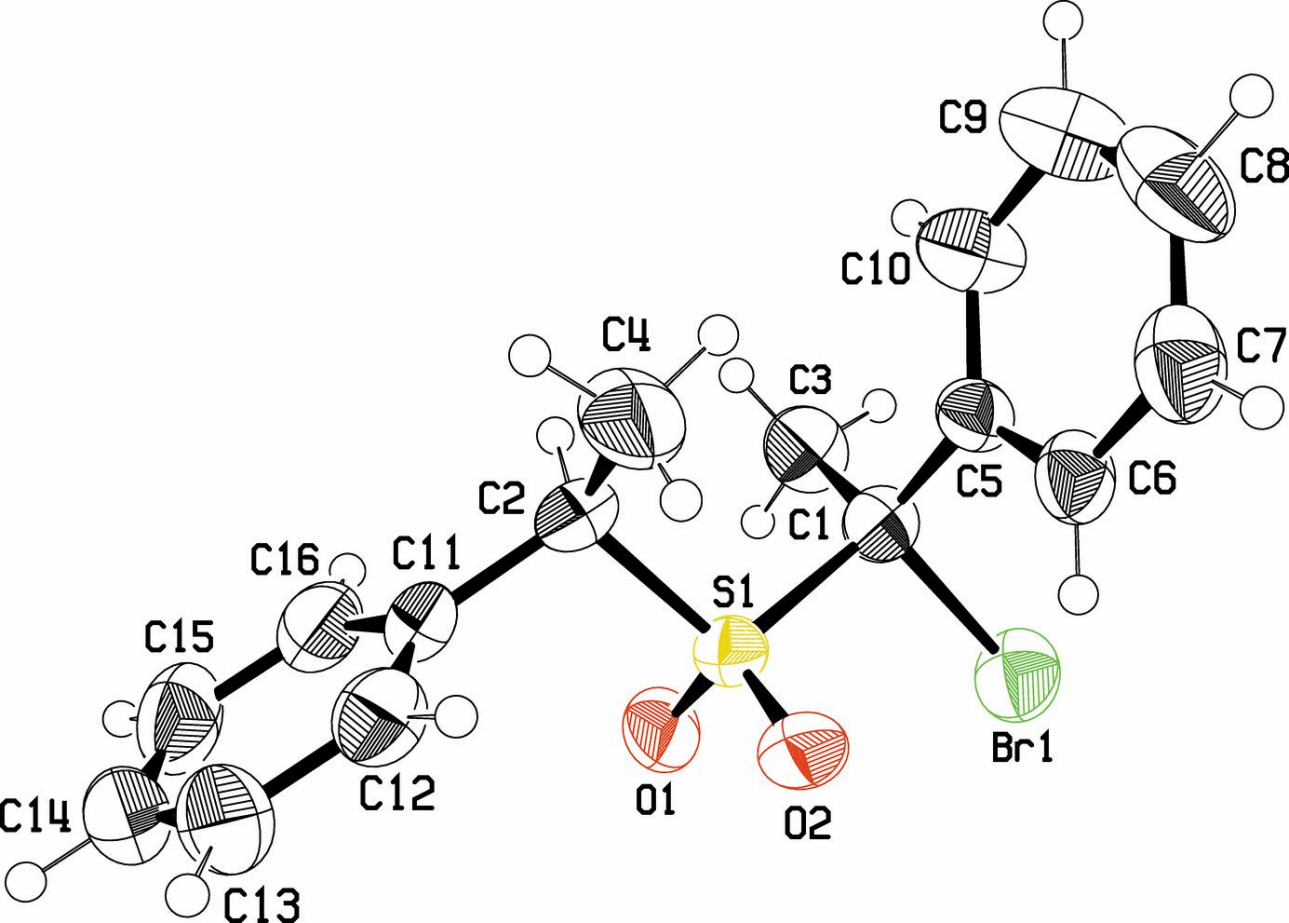
View of the title mol­ecule showing the atomic numbering and displacement ellipsoids at the 50% probability level.

The structure of the mol­ecule, with displacement ellipsoids, is shown in Fig. 1 ▸, where it is evident that the stereochemistries of the two α-C atoms to the sulfone group are *RR*. As the sample was present as a racemic mixture, there are equal numbers of mol­ecules in the crystal with the *SS* configuration – these configurations are referred to as *erythro* in the 1970 publication (Bordwell *et al.*, 1970 ▸). While the phenyl group C11–C16 is *trans* to the S1—C1 bond in the mol­ecule, phenyl group C5–C10 is *gauche* to the S1—C2 bond, with the Br1 atom taking the *trans* position. The planes of the two phenyl groups are inclined at 49.4 (2)° with one another.

The S=O distances of 1.426 (3) and 1.436 (4) Å are close to the mean of 1.437 Å found for 1142 sulfones with tetra­hedral α-C atoms in the Cambridge Structural Database (CSD; Groom *et al.*, 2016 ▸). The C1—Br1 bond length in the present structure is 1.976 (5) Å, close to the mean of 1.950 (2) Å found for 11000 aliphatic C—Br bond lengths in the database. The only other sulfone in the database with a phenyl group on each α-C atom and a bromine atom on at least one of the α-C atoms is entry WAVWOJ (Corfield, 2022 ▸). That analysis resulted from a similar collaboration with the Bordwell laboratory.

**Figure 2:**
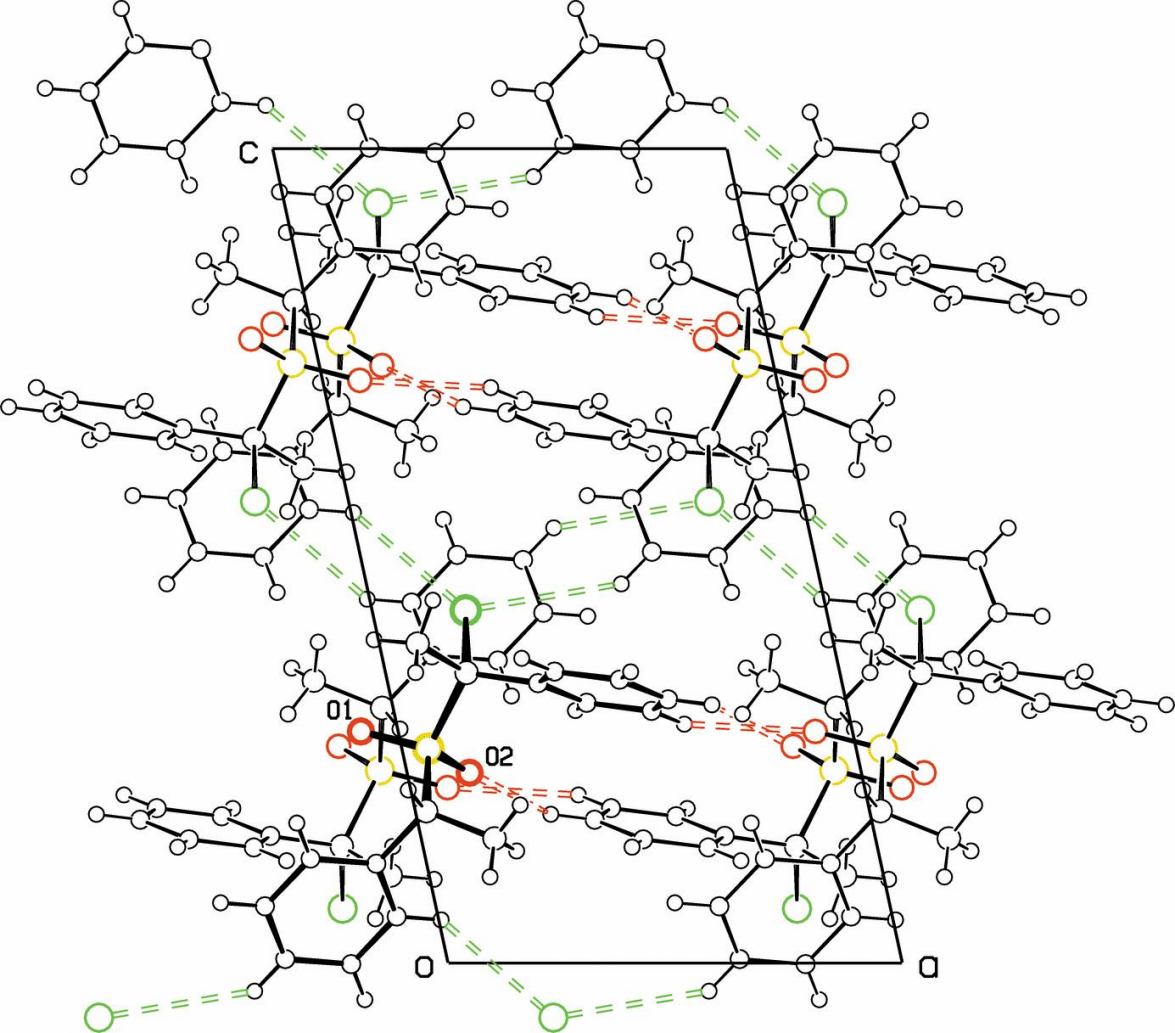
Projection of the crystal structure down the *b* axis. Atom colors: Br green, S yellow, O red, C,H black. C—H⋯Br and C—H⋯O hydrogen bonds are shown in green and red, respectively. The reference mol­ecule is bolded, with O1 and O2 labeled.

Table 1 ▸ lists four C—H⋯O and C—H⋯Br hydrogen bonds, chosen for contacts with C⋯O and C⋯Br distances close to the sum of the van der Waals radii and with C—H⋯O and C—H⋯Br angles of 140° or larger. These hydrogen bonds are shown in Fig. 2 ▸. The C—H⋯Br and C—H⋯O1 hydrogen bonds link the mol­ecules into sheets parallel to the *ab* plane, while the C—H⋯O2 hydrogen bonds complete the tri-periodic inter­molecular network *via* hydrogen bonds to mol­ecules related by a screw axis.

**Figure 3:**
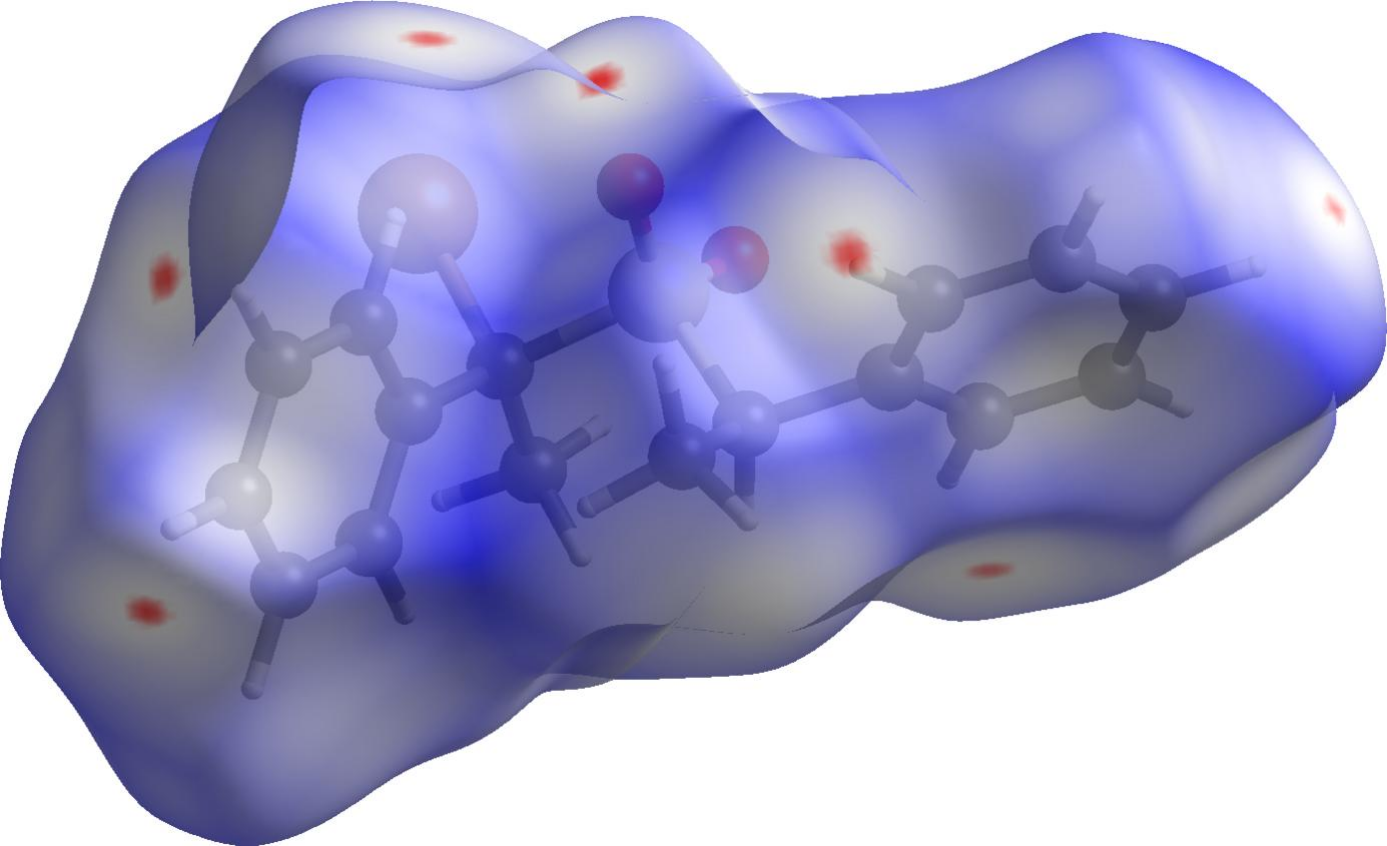
Hirshfeld *d*_norm_ surface for the title compound.

Analysis of the Hirshfeld surface of the mol­ecule carried out with *CrystalExplorer* (Spackman *et al.*, 2021 ▸) confirmed that the hydrogen bonds are the most significant inter­molecular contacts. The *d*_norm_ surface shown in Fig. 3 ▸ is colored blue for points where closest contacts are greater than the sum of the relevant van der Waals radii, while the red areas correspond to contacts closer than that sum. In the view shown, there are red areas corresponding to inter­molecular contacts for all of the four C—H donors and for two of the acceptors. There are also C⋯H contacts of 3.4–3.5 Å between phenyl rings C5–C10 related by the screw axes, which may be reflected in the red area at the lower right of Fig. 3 ▸. There are, however, no C⋯C contacts less than 4.0 Å between these screw-related phenyl rings.

## Synthesis and crystallization

The diastereomer was obtained by bromination of dl-bis-α-methyl­benzyl sulfone with *N*-bromo­succinimide. Details of similar syntheses by the Bordwell group are given in Carpino *et al.* (1971 ▸).

## Refinement

Crystal data, data collection and structure refinement details are summarized in Table 2 ▸. The data were collected in 1969 with a linear diffractometer unit. The (4 5 6) reflection was omitted due to a clear typewriter error in the data listing. Frequent system errors were common at that time, so that data collection could take much more time than is usual with today’s equipment. This is why the data do not reach the resolution expected in today’s work and why almost no symmetry equivalents were collected. No absorption corrections were made when the data was first processed, but the use of *XABS2* (Parkin *et al.*, 1995 ▸) in our current final refinements led to a smoother final difference map and somewhat lower reliability factors. *XABS2* rescales the observed data, using a tensor analysis. In Table 2 ▸, the minimum and maximum *XABS2* corrections of 0.84 and 1.12 for the transmission coefficients have been multiplied by exp (–*μ*r), with *μ* = 4.754 mm^−1^ and *r* = 0.23 mm.

The phenyl groups were refined as rigid hexa­gons, in order to reduce the number of parameters varied. C—C distances of 1.38 Å were chosen to minimize the reliability factors. C—H distances were constrained at 0.98 Å for the methine C2 atom, 0.96 Å for the methyl groups at C3 and C4, and 0.93 Å for the phenyl H atoms, while the H atom displacement parameters were set at 1.2*U*_eq_ of the parental C atoms.

## Supplementary Material

Crystal structure: contains datablock(s) I. DOI: 10.1107/S2414314624001895/wm4209sup1.cif

Structure factors: contains datablock(s) I. DOI: 10.1107/S2414314624001895/wm4209Isup2.hkl

Supporting information file. DOI: 10.1107/S2414314624001895/wm4209Isup3.cml

CCDC reference: 2335505

Additional supporting information:  crystallographic information; 3D view; checkCIF report

## Figures and Tables

**Table 1:** Hydrogen-bond geometry (Å, °)

*D*—H⋯*A*	*D*—H	H⋯*A*	*D*⋯*A*	*D*—H⋯*A*
C7—H7⋯O1^i^	0.93	2.67	3.468 (4)	145
C8—H8⋯O2^ii^	0.93	2.67	3.483 (4)	147
C12—H12⋯Br1^iii^	0.93	3.01	3.795 (3)	143
C14—H14⋯Br1^iv^	0.93	3.19	3.967 (3)	143

Symmetry codes: (i) 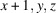 ; (ii) 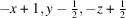 ; (iii) 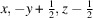 ; (iv) 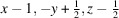 .

**Table 2:** Experimental details

Crystal data	
Chemical formula	C_16_H_17_BrO_2_S
*M*_r_	353.26
Crystal system, space group	Monoclinic, *P*2_1_/*c*
Temperature (K)	295
*a*, *b*, *c* (Å)	9.1051 (13), 10.665 (2), 16.688 (3)
β (°)	102.16 (2)
*V* (Å^3^)	1584.1 (5)
*Z*	4
Radiation type	Cu *K*α
μ (mm^−1^)	4.75
Crystal size (mm)	0.50 × 0.13 × 0.05

Data collection	
Diffractometer	Picker 4-circle diffractometer
Absorption correction	Empirical (using intensity measurements); four-dimensional tensor analysis (Parkin *et al.*, 1995 ▸)
*T*_min_, *T*_max_	0.28, 0.38
No. of measured, independent and observed [*I* > 2σ(*I*)] reflections	1821, 1678, 1373
*R*_int_	0.012
θ_max_ (°)	50.8
(sin θ/λ)_max_ (Å^−1^)	0.503

Refinement	
*R*[*F* ^2^ > 2σ(*F* ^2^)], *wR*(*F* ^2^), *S*	0.038, 0.103, 1.03
No. of reflections	1678
No. of parameters	159
H-atom treatment	H-atom parameters constrained
Δρ_max_, Δρ_min_ (e Å^−3^)	0.40, −0.32

Data reduction followed procedures in Corfield *et al.* (1973 ▸) with *p* = 0.06. Computer programs: Local Programs (Corfield & Gainsford, 1972 ▸), *SHELXL* (Sheldrick, 2015 ▸), *ORTEPIII* (Burnett & Johnson, 1996 ▸), *ORTEP-3 for Windows* (Farrugia, 2012 ▸), and *publCIF* (Westrip, 2010 ▸).

## full crystallographic data

### Crystal data

**Table:**

C_16_H_17_BrO_2_S	*D*_x_ = 1.481 Mg m^−^^3^
*M**_r_* = 353.26	Melting point: 385 K
Monoclinic, *P*2_1_/*c*	Cu *K*α radiation, λ = 1.5405 Å
*a* = 9.1051 (13) Å	Cell parameters from 7 reflections
*b* = 10.665 (2) Å	θ = 22.1–43.1°
*c* = 16.688 (3) Å	µ = 4.75 mm^−^^1^
β = 102.16 (2)°	*T* = 295 K
*V* = 1584.1 (5) Å^3^	Block, colorless
*Z* = 4	0.50 × 0.13 × 0.05 mm
*F*(000) = 720	

### Data collection

**Table:**

Picker 4-circle diffractometer	1373 reflections with *I* > 2σ(*I*)
Radiation source: sealed X-ray tube	*R*_int_ = 0.012
Oriented graphite 200 reflection monochromator	θ_max_ = 50.8°, θ_min_ = 5.0°
θ/2θ scans	*h* = 0→9
Absorption correction: empirical (using intensity measurements) Four-dimensional tensor analysis (Parkin *et al.*, 1995)	*k* = 0→10
*T*_min_ = 0.28, *T*_max_ = 0.38	*l* = −16→16
1821 measured reflections	3 standard reflections every 150 reflections
1678 independent reflections	intensity decay: 7(4)

### Refinement

**Table:**

Refinement on *F*^2^	Primary atom site location: heavy-atom method
Least-squares matrix: full	Secondary atom site location: difference Fourier map
*R*[*F*^2^ > 2σ(*F*^2^)] = 0.038	Hydrogen site location: inferred from neighbouring sites
*wR*(*F*^2^) = 0.103	H-atom parameters constrained
*S* = 1.03	*w* = 1/[σ^2^(*F*_o_^2^) + (0.*P*)^2^ + 1.890*P*] where *P* = (*F*_o_^2^ + 2*F*_c_^2^)/3
1678 reflections	(Δ/σ)_max_ < 0.001
159 parameters	Δρ_max_ = 0.40 e Å^−^^3^
0 restraints	Δρ_min_ = −0.32 e Å^−^^3^

### Special details

**Table:**

Geometry. All esds (except the esd in the dihedral angle between two l.s. planes) are estimated using the full covariance matrix. The cell esds are taken into account individually in the estimation of esds in distances, angles and torsion angles; correlations between esds in cell parameters are only used when they are defined by crystal symmetry. An approximate (isotropic) treatment of cell esds is used for estimating esds involving l.s. planes.
Refinement. At the time when this dataset was collected, mechanical failures were frequent enough that minimum redundancy was sought. This accounts for the low resolution of the data and the lack of many symmetry equivalents.

### Fractional atomic coordinates and isotropic or equivalent isotropic displacement parameters (Å^2^)

**Table:**

	*x*	*y*	*z*	*U*_iso_*/*U*_eq_	
Br1	0.20607 (7)	0.25910 (6)	0.43308 (4)	0.0668 (3)	
S1	0.05864 (13)	0.18503 (11)	0.26327 (7)	0.0437 (4)	
O1	−0.0819 (4)	0.2157 (3)	0.2845 (2)	0.0587 (10)	
O2	0.1378 (4)	0.2843 (3)	0.2337 (2)	0.0546 (9)	
C1	0.1784 (5)	0.1156 (5)	0.3565 (3)	0.0478 (13)	
C2	0.0240 (5)	0.0609 (5)	0.1867 (3)	0.0512 (14)	
H2	−0.005395	−0.014850	0.212602	0.061*	
C3	0.0847 (6)	0.0181 (5)	0.3923 (3)	0.0600 (15)	
H3A	−0.009418	0.054726	0.397050	0.072*	
H3B	0.138733	−0.007678	0.445547	0.072*	
H3C	0.066447	−0.053491	0.356734	0.072*	
C4	0.1613 (6)	0.0309 (6)	0.1531 (4)	0.080 (2)	
H4A	0.233922	−0.011531	0.194211	0.096*	
H4B	0.204106	0.107184	0.137720	0.096*	
H4C	0.133361	−0.022196	0.105833	0.096*	
C5	0.3308 (3)	0.0729 (3)	0.3438 (2)	0.0449 (13)	
C6	0.4283 (4)	0.1569 (3)	0.3197 (2)	0.0505 (13)	
H6	0.400215	0.240310	0.310129	0.061*	
C7	0.5676 (3)	0.1172 (4)	0.3097 (2)	0.0665 (16)	
H7	0.633361	0.173769	0.293460	0.080*	
C8	0.6093 (3)	−0.0066 (4)	0.3239 (2)	0.083 (2)	
H8	0.703208	−0.033387	0.317177	0.100*	
C9	0.5118 (5)	−0.0906 (3)	0.3480 (3)	0.089 (2)	
H9	0.539910	−0.174004	0.357563	0.107*	
C10	0.3725 (4)	−0.0509 (3)	0.3580 (2)	0.0685 (17)	
H10	0.306764	−0.107466	0.374233	0.082*	
C11	−0.1105 (3)	0.1051 (3)	0.12212 (18)	0.0447 (13)	
C12	−0.0907 (3)	0.1791 (3)	0.0573 (2)	0.0553 (14)	
H12	0.005339	0.203684	0.053144	0.066*	
C13	−0.2136 (5)	0.2166 (3)	−0.00117 (18)	0.0706 (17)	
H13	−0.200293	0.266399	−0.044821	0.085*	
C14	−0.3562 (4)	0.1801 (4)	0.0051 (2)	0.0786 (19)	
H14	−0.438943	0.205308	−0.034375	0.094*	
C15	−0.3759 (3)	0.1061 (4)	0.0698 (3)	0.0762 (19)	
H15	−0.471962	0.081501	0.074037	0.091*	
C16	−0.2530 (4)	0.0686 (3)	0.1284 (2)	0.0607 (15)	
H16	−0.266331	0.018783	0.172003	0.073*	

### Atomic displacement parameters (Å^2^)

**Table:**

	*U*^11^	*U*^22^	*U*^33^	*U*^12^	*U*^13^	*U*^23^
Br1	0.0632 (4)	0.0751 (5)	0.0627 (4)	0.0016 (3)	0.0149 (3)	−0.0201 (3)
S1	0.0398 (7)	0.0383 (7)	0.0520 (8)	0.0021 (6)	0.0073 (6)	−0.0049 (6)
O1	0.039 (2)	0.065 (2)	0.070 (2)	0.0087 (18)	0.0075 (18)	−0.0142 (19)
O2	0.056 (2)	0.038 (2)	0.068 (2)	−0.0030 (17)	0.0089 (18)	0.0074 (17)
C1	0.049 (3)	0.046 (3)	0.048 (3)	−0.001 (3)	0.008 (2)	0.000 (3)
C2	0.060 (3)	0.034 (3)	0.056 (3)	0.001 (3)	0.006 (3)	−0.010 (2)
C3	0.051 (3)	0.063 (4)	0.069 (4)	−0.007 (3)	0.018 (3)	0.016 (3)
C4	0.069 (4)	0.091 (5)	0.075 (4)	0.035 (4)	0.005 (3)	−0.028 (4)
C5	0.041 (3)	0.046 (3)	0.047 (3)	0.008 (3)	0.007 (2)	−0.005 (2)
C6	0.037 (3)	0.059 (3)	0.056 (3)	−0.003 (3)	0.012 (2)	−0.007 (3)
C7	0.045 (4)	0.088 (5)	0.066 (4)	−0.005 (3)	0.013 (3)	−0.011 (3)
C8	0.055 (4)	0.118 (6)	0.077 (4)	0.035 (4)	0.015 (3)	−0.002 (4)
C9	0.089 (5)	0.080 (5)	0.102 (5)	0.037 (4)	0.027 (4)	0.016 (4)
C10	0.067 (4)	0.060 (4)	0.083 (4)	0.015 (3)	0.026 (3)	0.012 (3)
C11	0.047 (3)	0.039 (3)	0.045 (3)	−0.005 (2)	0.004 (2)	−0.010 (3)
C12	0.057 (3)	0.056 (3)	0.054 (4)	−0.004 (3)	0.015 (3)	−0.007 (3)
C13	0.090 (5)	0.075 (4)	0.042 (3)	0.004 (4)	0.002 (3)	0.002 (3)
C14	0.080 (5)	0.072 (4)	0.069 (4)	0.020 (4)	−0.018 (4)	−0.015 (4)
C15	0.049 (4)	0.087 (5)	0.088 (5)	−0.011 (3)	0.003 (4)	−0.025 (4)
C16	0.070 (4)	0.053 (3)	0.058 (4)	−0.011 (3)	0.010 (3)	−0.008 (3)

### Geometric parameters (Å, º)

**Table:**

Br1—C1	1.976 (5)	C6—H6	0.9300
S1—O2	1.426 (3)	C7—C8	1.3800
S1—O1	1.436 (4)	C7—H7	0.9300
S1—C2	1.820 (5)	C8—C9	1.3800
S1—C1	1.856 (5)	C8—H8	0.9300
C1—C5	1.516 (5)	C9—C10	1.3800
C1—C3	1.543 (7)	C9—H9	0.9300
C2—C4	1.509 (7)	C10—H10	0.9300
C2—C11	1.525 (5)	C11—C12	1.3800
C2—H2	0.9800	C11—C16	1.3800
C3—H3A	0.9600	C12—C13	1.3800
C3—H3B	0.9600	C12—H12	0.9300
C3—H3C	0.9600	C13—C14	1.3800
C4—H4A	0.9600	C13—H13	0.9300
C4—H4B	0.9600	C14—C15	1.3800
C4—H4C	0.9600	C14—H14	0.9300
C5—C6	1.3800	C15—C16	1.3800
C5—C10	1.3800	C15—H15	0.9300
C6—C7	1.3800	C16—H16	0.9300
			
O2—S1—O1	117.2 (2)	C5—C6—C7	120.0
O2—S1—C2	108.8 (2)	C5—C6—H6	120.0
O1—S1—C2	107.9 (2)	C7—C6—H6	120.0
O2—S1—C1	109.6 (2)	C8—C7—C6	120.0
O1—S1—C1	106.3 (2)	C8—C7—H7	120.0
C2—S1—C1	106.4 (2)	C6—C7—H7	120.0
C5—C1—C3	116.7 (4)	C7—C8—C9	120.0
C5—C1—S1	113.3 (3)	C7—C8—H8	120.0
C3—C1—S1	108.6 (3)	C9—C8—H8	120.0
C5—C1—Br1	109.1 (3)	C10—C9—C8	120.0
C3—C1—Br1	106.1 (3)	C10—C9—H9	120.0
S1—C1—Br1	101.8 (2)	C8—C9—H9	120.0
C4—C2—C11	114.0 (4)	C9—C10—C5	120.0
C4—C2—S1	112.4 (4)	C9—C10—H10	120.0
C11—C2—S1	105.4 (3)	C5—C10—H10	120.0
C4—C2—H2	108.3	C12—C11—C16	120.0
C11—C2—H2	108.3	C12—C11—C2	120.8 (3)
S1—C2—H2	108.3	C16—C11—C2	119.2 (3)
C1—C3—H3A	109.5	C13—C12—C11	120.0
C1—C3—H3B	109.5	C13—C12—H12	120.0
H3A—C3—H3B	109.5	C11—C12—H12	120.0
C1—C3—H3C	109.5	C12—C13—C14	120.0
H3A—C3—H3C	109.5	C12—C13—H13	120.0
H3B—C3—H3C	109.5	C14—C13—H13	120.0
C2—C4—H4A	109.5	C13—C14—C15	120.0
C2—C4—H4B	109.5	C13—C14—H14	120.0
H4A—C4—H4B	109.5	C15—C14—H14	120.0
C2—C4—H4C	109.5	C16—C15—C14	120.0
H4A—C4—H4C	109.5	C16—C15—H15	120.0
H4B—C4—H4C	109.5	C14—C15—H15	120.0
C6—C5—C10	120.0	C15—C16—C11	120.0
C6—C5—C1	120.7 (3)	C15—C16—H16	120.0
C10—C5—C1	119.3 (3)	C11—C16—H16	120.0
			
O2—S1—C1—C5	−54.5 (4)	C10—C5—C6—C7	0.0
O1—S1—C1—C5	177.9 (3)	C1—C5—C6—C7	178.9 (3)
C2—S1—C1—C5	63.0 (4)	C5—C6—C7—C8	0.0
O2—S1—C1—C3	174.1 (3)	C6—C7—C8—C9	0.0
O1—S1—C1—C3	46.5 (4)	C7—C8—C9—C10	0.0
C2—S1—C1—C3	−68.3 (4)	C8—C9—C10—C5	0.0
O2—S1—C1—Br1	62.5 (3)	C6—C5—C10—C9	0.0
O1—S1—C1—Br1	−65.1 (3)	C1—C5—C10—C9	−178.9 (3)
C2—S1—C1—Br1	−180.0 (2)	C4—C2—C11—C12	−36.1 (5)
O2—S1—C2—C4	45.6 (5)	S1—C2—C11—C12	87.6 (3)
O1—S1—C2—C4	173.8 (4)	C4—C2—C11—C16	142.9 (4)
C1—S1—C2—C4	−72.5 (5)	S1—C2—C11—C16	−93.3 (3)
O2—S1—C2—C11	−79.2 (3)	C16—C11—C12—C13	0.0
O1—S1—C2—C11	49.0 (4)	C2—C11—C12—C13	179.0 (3)
C1—S1—C2—C11	162.7 (3)	C11—C12—C13—C14	0.0
C3—C1—C5—C6	−173.6 (3)	C12—C13—C14—C15	0.0
S1—C1—C5—C6	59.2 (4)	C13—C14—C15—C16	0.0
Br1—C1—C5—C6	−53.5 (4)	C14—C15—C16—C11	0.0
C3—C1—C5—C10	5.3 (5)	C12—C11—C16—C15	0.0
S1—C1—C5—C10	−121.9 (3)	C2—C11—C16—C15	−179.0 (3)
Br1—C1—C5—C10	125.4 (3)		

### Hydrogen-bond geometry (Å, º)

**Table:**

*D*—H···*A*	*D*—H	H···*A*	*D*···*A*	*D*—H···*A*
C7—H7···O1^i^	0.93	2.67	3.468 (4)	145
C8—H8···O2^ii^	0.93	2.67	3.483 (4)	147
C12—H12···Br1^iii^	0.93	3.01	3.795 (3)	143
C14—H14···Br1^iv^	0.93	3.19	3.967 (3)	143

Symmetry codes: (i) *x*+1, *y*, *z*; (ii) −*x*+1, *y*−1/2, −*z*+1/2; (iii) *x*, −*y*+1/2, *z*−1/2; (iv) *x*−1, −*y*+1/2, *z*−1/2.
